# Genetic polymorphism and expression of HSF1 gene is significantly associated with breast cancer in Saudi females

**DOI:** 10.1371/journal.pone.0193095

**Published:** 2018-03-01

**Authors:** Sahar Almotwaa, Mohamed Elrobh, Huda AbdulKarim, Mohamed Alanazi, Sooad Aldaihan, Jilani Shaik, Maha Arafa, Arjumand Sultan Warsy

**Affiliations:** 1 Department of Biochemistry, College of Science, King Saud University, Riyadh, Saudi Arabia; 2 Department of Biochemistry, College of Science, King Saud University, Riyadh, Saudi Arabia; 3 Head of the Hematology/Oncology Unit at King Fahad Medical City Hospital, Comprehensive Cancer Center, Riyadh, Saudi Arabia; 4 Department of Biochemistry, College of Science, King Saud University, Riyadh, Saudi Arabia; 5 Department of Biochemistry, College of Science, King Saud University, Riyadh, Saudi Arabia; 6 Genome Research Chair, Department of Biochemistry, College of Science, King Saud University, Riyadh, Saudi Arabia; 7 Department of Pathology, College of Medicine, King Saud University, Riyadh, Saudi Arabia; 8 Senior Scientist, Central Laboratory, Center for Science and Medical Studies for Girls, King Saud University, Riyadh, Saudi Arabia; University of Wisconsin Madison, UNITED STATES

## Abstract

The transcription factor, heat shock factor 1 (HSF1), influences the expression of heat shock proteins as well as other activities like the induction of tumor suppressor genes, signal transduction pathway, and glucose metabolism. We hypothesized that single nucleotide polymorphisms (SNPs) in HSF1 gene might affect its expression or function which might have an influence on the development of breast cancer. The study group included 242 individuals (146 breast cancer patients and 96 healthy controls). From the cancer patients, genomic DNA was extracted from 96 blood samples and 50 Formalin-Fixed Paraffin Embedded (FFPE) tissues, while from the controls DNA were extracted from blood only. Genotype was carried out for four SNPs in the HSF1 gene (rs78202224, rs35253356, rs4977219 and rs34404564) using Taqman genotyping assay method. The HSF1 expression was investigated using immunohistochemistry on FFPE tissues (cancer tissue and adjacent normal tissue). The SNP rs78202224 (G>T) was significantly associated with increased risk of breast cancer. The combined TT + GT genotype (OR: 6.91; p: 0.035) and the T allele showed high risk (OR: 5.81; p:0.0085) for breast cancer development. The SNP rs34404564 (A>G) had a protective effect against the development of breast cancer. The genotype AG (OR: 0.41; p = 0.0059) and GG+AG (OR: 0.52; p: 0.026) occurred at a significantly lower frequency in the breast cancer patients compared to the frequency in healthy controls. No significant relationship was identified between either rs35253356 (A>G) or rs4977219 (A>C) and breast cancer in Saudi. The HSF1 protein expression was higher in all invasive and in situ breast carcinoma compared to the normal tissue. A stronger positive staining for HSF1 was found in the nucleus compared to the cytoplasm. Our results show that HSF1 gene expression is elevated in breast cancer tissue and two of the studied SNPs correlate significantly with cancer development.

## Introduction

Breast cancer is the most frequently encountered amongst females and is a leading cause of cancer-related deaths all over the world and in Saudi Arabia [1]. Heat Shock Factor 1 enhances the survival, spread, and proliferation of malignant cells [2]. It is considered as a guardian of proteostasis, a phenomenon shown to be of significance in cancer cells [3]. Sentagata et al. [4] demonstrated that nuclear HSF1 levels increase in 80% of *in situ* and invasive breast carcinomas. HSF1 expression was linked to high histologic grade, larger tumor size, and nodal involvement at diagnosis in invasive carcinomas. High HSF1 levels were associated with increased level of mortality. It was shown that HSF1 is involved in both progression and suppression of breast cancer due to its effect on tumor suppressor gene (p53), oncogene RAS60 and human epidermal growth factor receptor 2 (HER2) [2, 5–8]. Sentagata et al. [4] suggested that HSF1 may be a useful therapeutic target for cancer.

The HSP levels become elevated in a wide spectrum of malignant cells including mammary carcinoma cells [9]. Transcription of *HSPs* genes is regulated by HSF1 that senses cellular exposure to stress and turns on rapid induction of HSPs [10]. The *HSF1* activation in breast cancer cell line (MSF7) was shown to cause elevated expression of HSP60, HSP70 and HSP90 and the cancer cells escaped from apoptotic cell death [11, 12]. Sarkar et al. [13] showed that it is the down-regulation of *HSF1*, *HSP90* and *HSP70* expression in breast cancer cell line MCF-7 and MDA-MB-231 cell, which results in inducing apoptosis. HSP90 holds the main responsibility of enhancing the spread of tumor via chaperoning the oncogenes that have mutated and over-expressed. It has a positive influence on transformation and progression of tumors [9, 14]. Yamaki et al. [15], Tsutsumi et al. [16] and Sims et al. [17] have demonstrated that the inhibition of HSP90 and HSP70 lowers the migration of cancer cells and their invasive ability. The HSF1 is a monomer in its inactive state, and upon the cells exposed to the stress conditions, it is homo trimerized through four leucine zipper domains (LZ1-4). The LZ1 to LZ3 domains are located in the N-terminal while LZ4 domain is on the C-terminal [18].

This study was based on the hypothesis that 'genetic polymorphism in HSF1 gene might interfere with its activity and hence influence the development of breast cancer'. Since very few studies are reported in literature on the association between polymorphisms in HSF1 and risk of colon cancer, the SNPs were randomly selected, based on their location in HSF1 gene to investigate the role of polymorphisms in the generation of breast cancer susceptibility in Saudi breast cancer patients. Of the studied SNPs, rs78202224 (G>T), is a non-synonymous mutation, located in exon 9; and rs35253356 (A>G), rs4977219 (A>C) and rs34404564 (A>G) located in intron 1. Qualitative levels of HSF1 were determined by immunohistochemistry to identify the interaction between HSF1 and breast cancer to correlate *HSF1* expression and selected polymorphic sites with the clinical presentation in Saudi females.

## Materials and methods

### Study population

The study was approved by the Institutional Review Board of King Khalid Hospital (IRB No. 15-089E). Only those females were included who volunteered to enroll in the study and a written informed consent was obtained from all participants. (The details of age of each patient and control is presented in Supporting Information files entitled “All data HSF paper” Table A in S1 File). Blood samples were obtained from patients and normal healthy controls, attending clinics of the Clinical co-investigator at King Fahad Medical City (following approval of the IRB) and collected in ethylene-diamine tetra acetic acid (EDTA) tubes via venipuncture from 96 breast cancer patients (S1 File, Cancer blood) and 96 controls with no personal or family history of breast cancer (Table B in S1 File, Control blood). All controls were age matched and recruited following physical examinations after diagnostic exclusion of cancer and cancer-related disease. Demographic data and clinical data of the patients was collected from their files and entered on special spreadsheets, after obtaining informed and written consent from each patient. Formalin-Fixed Paraffin Embedded (FFPE) tissues of the breast cancer patients, enrolled in this study were obtained from the Pathology Department at the King Khalid University Hospital, KSU, Riyadh. The FFPE were from 50 normal and 50 cancer tissues from the same patient (Table C in S1 File, Tissue).

### Genomic DNA extraction and quantification

Three ml blood was extracted in EDTA tubes for the extraction of genomic DNA using kits from Gentra Puregene Systems Inc., USA. The protocol used for the extraction of DNA from the FFPE tissues was provided by the manufacturer (QIAamp DNA FFPE Tissue, USA), with certain modifications as described by Sam et. al. [19]. Quantification of DNA was carried out by measuring absorbance using NanoDrop Lite Spectrophotometer (Thermo Scientific, USA).

### TaqMan genotyping assay by real time PCR (qPCR)

Genotyping was conducted on Real-time PCR (qPCR). Single nucleotide polymorphisms (rs78202224, rs35253356, rs4977219, and rs34404564 of the *HSF1* gene) were assessed with the LightCycler 480 Instrument II Real-Time PCR System (Roche Applied Science, Indianapolis, USA) using the TaqMan genotyping assay (Life Technologies, Foster City, CA, USA). The final volume for each reaction was 10.8 μL, containing 5.5 μL TaqMan Genotyping Master Mix, 0.27 μL TaqMan probe mix, 3 μL DNase/RNase-Free distilled water (Bio Basic Inc. CA) and 2 μL genomic DNA. The real-time PCR steps included an initial activation step at 95°C for 10 min, followed by 40 cycles for genomic DNA from blood and 60 cycles for DNA extracted from tissues sample at of 92°C for 15 sec., and 60°C for 1 min. To validate results from real-time PCR, around five percent of assays were repeated.

### Immunohistochemistry

Heat shock factor 1 (HSF1) expression was studied in 14 invasive ductal carcinomas and one invasive lobular carcinoma (Table D in S1 file, Tissue Samples). Tissue sections of 3-micron thickness were cut into coated slides by using Leica RM2235 Rotary Microtome (Leica Biosystems, Wetzlar, Germany). Immunohistochemistry assay was conducted using anti-HSF1 rat monoclonal antibody (Thermo Scientific, CA, USA) with UltraView DAB Detection Kit (Ventana, Arizona, USA) on a BenchMark XT automated staining system (Ventana, Arizona, USA). The tissue stained with the HSF1 antibody and the immunoreaction was given a score according to signal intensity: 0, negative immune-staining; 1+, weakly positive immune-staining; 2+, moderately positive immune-staining; and 3+, strongly positive immune-staining. 0 was considered negative expression, 1+ was considered low HSF1 expression and 2+ and 3+ was considered high HSF1 expression. Interpretation of the immunohistochemistry data was performed by a specialized pathologist at KKUH.

### Statistical analysis

The Genotype and allele frequencies were subjected to test the Hardy-Weinberg equilibrium. Statistical computational software available at Institute fur Humangenetik (URL: https://ihg.gsf.de/cgi-bin/hw/hwa1.pl) were used to calculate the odds ratio (OR), 95% confidence intervals (CI), Chi-square (χ2) and p value for each genotype (and allele) to compare the significance of the difference between the patients and controls. The SPSS program (v20 for Windows) was used to conduct correlation studies between genotypes and clinical data. Differences with p values of less than 0.05 were considered as statistically significant.

## Results

A total of 146 women diagnosed with breast cancer were included in this study, with a median age of 49 years old. The anthropometric and clinical characteristics of the studied breast cancer patients are summarized in Table 1.

**Table 1:** Clinical characteristic of breast cancer patients.

Variable	Cases (n = 146) (%)
Age (years) Equal to or less than 48/More than 48	76 (52)/70 (48)
Tumor Grade Grade I/Grade II/Grade III	5(3.5)/76(54)/60 (42.5)
Estrogen Receptor (ER) ER-/ER+	55 (38)/91 (62)
Progesterone Receptor (PR) PR-/PR+	58 (40)/88 (60)
HER2 status HER2- /HER2+/ TNBC	87(60)/58(40)/28(19.2)

The four SNPs of HSF1 gene were genotyped in 146 breast cancer patients (include 50 tissue and 96 blood samples) and 96 healthy controls (blood samples). For each polymorphism, the Hardy-Weinberg equilibrium (HWE) was studied and the results are presented in Table 2. One SNP did not obey HWE, but all others were in equilibrium in the control group.

**Table 2:** Genotype distributions of four SNPs in HSF1 gene in the Saudi controls and patients according to the Hardy-Weinberg equilibrium (HWE).

Genotype	Case No.	HWE P-value	Control No.	HWE P-value
rs78202224				
GG	136	<0.001	94	<0.001
GT	3		0	
TT	7		1	
rs35253356				
GG	45	0.1700	24	0.680
AG	64		45	
AA	36		25	
rs4977219				
AA	29	0.398	23	0.224
AC	66		40	
CC	50		29	
rs34404564				
AA	56	0.001	24	0.269
AG	40		42	
GG	47		29	

Table 3 shows the genotype and allele frequencies of the four SNPs in HSF1 gene and their association with breast cancer risk. Among the breast cancer patients, the genotypes of the SNP rs78202224 (G/T) were as follows: 136 (93%) for GG, 3 (2%) for GT and 7 (4.79%) for TT. For the healthy control, the genotypic allelic distribution was 94 (98.95%) for GG, 0 for GT and 1 (1.05%) for TT. Combined TT and GT genotypes show a significant difference between breast cancer cases and healthy control (OR = 6.91, χ^2^ = 4.44, p = 0.035). In addition, the frequency of T allele was associated with higher risk of breast cancer development (OR = 5.81, χ^2^ = 6.91, p = 0.0085). The genotypic and allelic distribution for SNPs rs35253356 (G/A) and rs4977219 (A/C) did not show significant association when compared with healthy control (p > 0.05). Genotype frequencies of SNP rs34404564 (AA, AG, GG genotypes) in breast cancer cases were 56 (39.16%), 40 (27.97%) and 47 (32.87%), respectively, whereas as in healthy controls the frequencies were 24 (25.26%), 42 (44.21%) and 29 (30.53%), respectively. The frequency of heterozygous (AG) in patient samples showed a significant difference when compared with healthy controls (OR = 0.41, χ^2^ = 7.55, p = 0.0059). This difference indicates a protective effect of the AG genotype for breast cancer patient. The frequency of the combined GG and AG genotypes was also significantly different between breast cancer cases and healthy control (OR = 0.52 χ^2^ = 4.94, p = 0.026).

**Table 3:** Genotype and allele frequencies of four SNPs in HSF1 gene and association with breast cancer risk.

Variation	Case No. (%)	Control No. (%)	Breast cancer vs. Control			P-value
			OR	CI	*χ*^2^	
rs78202224						
GG	136 (93.15)	94 (98.95)	Ref.			
GT	3 (2.05)	0	4.85	0.25–94.91	2.06	0.15
TT	7 (4.79)	1 (1.05)	4.83	0.58–39.97	2.59	0.11
Total	146	95				
TT+GT	10	1	6.91	0.87–54.9	4.44	0.035*
G	275 (94.18)	188 (98.95)	Ref.			
T	17 (5.82)	2 (1.05)	5.81	1.32–25.4	6.91	0.0085*
Total	292	190				
rs35253356						
GG	45 (31.03)	24 (25.53)	Ref.			
AG	64 (44.14)	45 (47.87)	0.76	0.41–1.41	0.75	0.39
AA	36 (24.83)	25 (26.6)	0.77	0.377–1.56	0.53	0.47
Total	145	94				
AA+AG	100	70	0.76	0.42–1.36	0.84	0.36
G	154 (53.1)	93 (49.47)	Ref.			
A	136 (46.9)	95 (50.53)	0.86	0.59–1.24	0.6	0.44
Total	290	188				
rs4977219						
AA	29 (20)	23 (25)	Ref.			
AC	66 (45.52)	40 (43.48)	1.31	0.67–2.57	0.61	0.43
CC	50 (34.48)	29 (31.52)	1.37	0.67–2.79	0.74	0.40
Total	145	92				
AC+CC	116	69	1.33	0.71–2.49	0.82	0.36
A	124(42.76)	86 (46.74)	Ref.			
C	166(57.24)	98 (53.26)	1.17	0.81–1.7	0.72	0.39
Total	290	184				
rs34404564						
AA	56 (39.16)	24 (25.26)	Ref.			
AG	40 (27.97)	42 (44.21)	0.41	0.21–0.78	7.55	0.0059*
GG	47 (32.87)	29 (30.53)	0.69	0.36–1.35	1.16	0.28
Total	143	95				
GG+AG	87	71	0.52	0.3–0.93	4.94	0.026*
A	152 (53.15)	90 (47.37)	Ref.			
G	134 (46.85)	100(52.63)	0.40	0.55–1.15	1.53	0.22
Total	286	190				

* Statistically significant at p ≤ 0.05.

To determine if there was any difference in the frequency of the SNPs in the cancer tissues and the normal tissue, the genotyping results from the matched tissues (50 breast cancer tissues matched with 50 normal tissues) were compared. No statistically significant difference was observed between the two groups (p = 1) (results not shown).

### Association between age of onset of breast cancer and clinical characteristics with four SNPs in HSF1 gene

The correlation between risk of breast cancer in polymorphism of the four studied SNPs and the age of onset of breast cancer with the clinic-pathological characteristics was analyzed. The results were grouped according to age less than or equal to 48 years (n = 76) and age more than 48 years (n = 70). The distribution of the genotypes and alleles in HER2 negative (n = 87), HER2 positive (n = 58), ER negative (n = 55), ER-positive (n = 91), PR-negative (n = 58) and PR positive (n = 88) were separately compared with healthy control (n = 96). Summary of the results is present in Table 4.

**Table 4:** Summary table for genotype frequencies and P-value for SNPs in HSF1 gene and association with clinical characteristics.

SNPs	Variation	Age (Yrs) Equal to or less than 48	Age (Yrs) More than 49	HER2 -	HER2+	ER-	ER+	PR-	ER+
		P-Value							
rs78202224	GT		0.73						
	TT		0.29	0.074	0.28	0.036*	0.29	0.11	0.26
	T allele		0.21	0.0068*	0.061	0.001*	0.09	0.008*	0.093
rs35253356	AG	0.39	0.61	0.16	0.94	0.92	0.23	0.42	0.72
	AA	0.4	0.68	0.38	0.82	0.583	0.50	0.28	0.76
	A allele	0.375	0.67	0.32	0.81	0.58	0.46	0.26	0.75
rs4977219	AC	0.4	0.73	0.72	0.34	0.43	0.57	0.18	0.87
	CC	0.12	0.84	0.4	0.58	0.669	0.35	0.24	0.66
	C allele	0.12	0.72	0.35	0.64	0.71	0.33	0.29	0.64
rs34404564	AG	0.21	0.008*	0.016*	0.0178*	0.08	0.005*	0.14	0.003*
	GG	0.54	0.045*	0.5	0.20	0.489	0.27	0.83	0.148
	G allele	0.64	0.023*	0.47	0.14	0.457	0.20	0.88	0.08

* Statistically significant at p ≤ 0.05.

The SNP rs34404564 occurred at a significantly different frequency in the patients and controls when the results in the patient who was more than 48 years old were compared to those patients who were less than 48 years of age. This difference indicates a protective effect of AG and GG genotype and G allele for breast cancer patients above 48 years old.

The polymorphism of the SNP rs78202224 in HER2 negative patients showed significantly increased risk of breast cancer when compared with healthy controls at TT+GG genotype and T allele. In HER2 positive patients, it showed significant increase between breast cancer patients and healthy controls at TT+GG genotype. It also was significantly increased in breast cancer patients and healthy controls in ER-negative patients at TT and TT+GG genotype and T allele. In PR negative patients, a significantly increased risk of breast cancer was observed when compared with healthy controls at TT+GG genotype and T allele, whereas no significant association in breast cancer in ER positive and PR positive patients was observed when comparing breast cancer patients and healthy controls.

For the polymorphism of the SNP rs34404564, a significant decreased risk of breast cancer in HER2 negative patients when compared with healthy controls at AG genotype was shown. For the AG and GG+AG genotype and in patients with HER2 positive, ER positive and PR positive, a significant decrease risk of breast cancer was observed when compared with healthy controls while no significant association with ER negative and PR negative patients when compared with healthy control. On the other hand, no significant relationship between HER2+, ER+, and PR+ with the two SNPs; rs35253356 and rs4977219, genotypes under investigation in breast cancer patient and healthy individuals.

### Expression of HSF1

**Fig 1:**
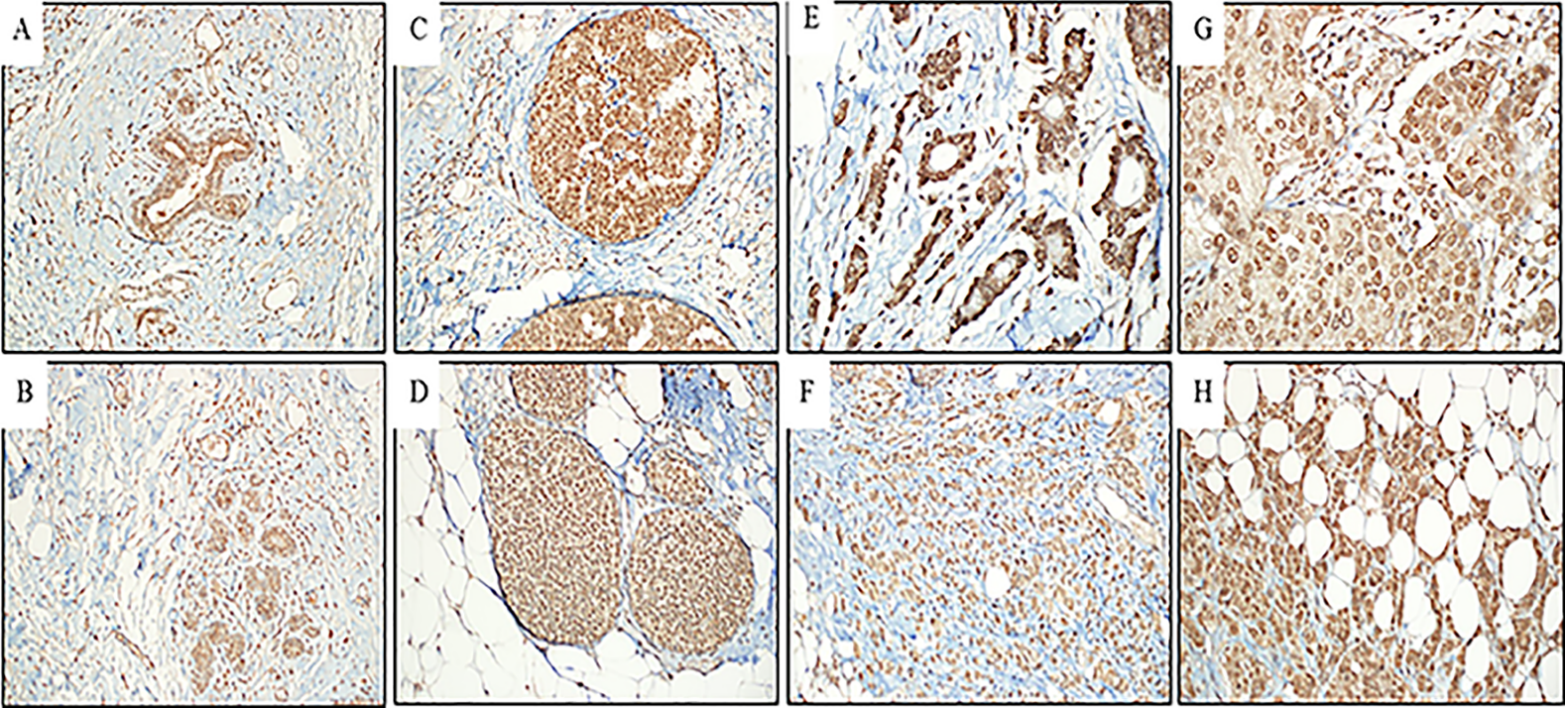
**HSF1 protein expression in breast cancer:** (A) normal ductal tissue visualized under a microscope with a digital zoom (micro, 200X), negative immune-staining; (B) normal lobular tissue visualized under a microscope with a digital zoom (micro, 200X), negative immune-staining; (C) ductal carcinoma *in situ* visualized under a microscope with a digital zoom (micro, 200X), strongly positive immune-staining (+3); (D) lobular carcinoma *in situ* visualized under a microscope with a digital zoom (micro, 200X), moderately positive immune-staining (+2); (E) invasive ductal carcinoma grade I visualized under a microscope with a digital zoom(micro, 400X), strongly positive immune-staining (+3); (F) invasive lobular carcinoma grade I visualized under a microscope with a digital zoom(micro, 200X), moderately immune-staining (+2); (G) invasive ductal carcinoma grade II visualized under a microscope with a digital zoom (micro, 400X), strongly positive immune-staining (+3); (H) invasive ductal carcinoma grade III between fatty tissue of the breast visualized under a microscope with a digital zoom (micro, 200X), strongly positive immune-staining (+3).

High protein expression was observed in all invasive breast cancer tissues relative to the normal tissue (Fig 1A and 1B). In the same fields of the breast cancer, patient ductal carcinoma in situ (DCIS) and lobular carcinoma in situ (LCIS) high expression levels were observed (Fig 1C and 1D). The HSF1 expression was high in patients with all histological grades of cancer. In breast cancer patient, the IHC staining were 27% (n = 4) in grade I, well differentiated (Fig 1E and 1F), 20% (n = 3) in grade II; moderately differentiated (Fig 1G), 53% (n = 8 in grade III; poorly differentiated (Fig 1H).

### Disease severity and genotypes of the four studied SNPs

The patients were grouped according to the severity of the disease presentations, as severe and intermediate severity, and the genotypes identified in each group were counted. The results are presented in Table 5.

**Table 5:** Prevalence of the genotypes of the four studied SNPs in patients with different clinical severity of the disease.

Clinical severity of Breast cancer	SNPs studied in HSF1 gene							
	rs78202224		rs35253356		rs4977219		rs34404564	
	Genotype	%	Genotype	%	Genotype	%	Genotype	%
High severity	GG	83.3	AA	33.3	AA	16.7	AA	50
	TT	16.7	AG	50	AC	50	AG	16.7
			GG	16.7	CC	33.3	GG	33.3
Intermediate severity	GG	77.8	AA	44.4	AA	33.3	AA	55.5
	TT	22.2	AG	11.1	AC	22.2	AG	0
			GG	44.4	CC	44.4	GG	44.4

## Discussion

In the recent years, considerable interest has been directed to the role of HSF1 in cancer development [20–25]. The significance of HSF1 gene in cancer development is becoming apparent since it influences expression of HSPs, tumor suppressor genes, oncogenes, signal transduction and metabolism of glucose. It results in enhancing invasion, survival, transformation and progression of tumor cell [2,4, 5–8, 11–17, 26]. Hence, it is regarded as a powerful multifaceted facilitator of oncogenesis [22]. The results of this study show that rs78202224 (G>T), a missense transversion mutation of the amino acid 365 located adjacent to leucine zipper (LZ3) trimerization domain, results in the substitution of proline (an imino acid) by threonine (a hydroxyl-containing amino acid) that is associated with significantly increased risk of breast cancer when compared with healthy controls. The combined TT + GT genotypes and the T allele showed high risk (p-value < 0.05) for breast cancer development and the allele frequencies were significantly different. On the other hand, when compared between the breast cancer patients and the control group for rs35253356 (G>A) and rs4977219 (A>C), both located in intron 1, it did not show any significant association with breast cancer. The mutated allele in the SNP rs34404564 (A>G) also located in intron 1 showed a protective effect against the development of breast cancer. AG and combined GG+AG genotypes showed a statistically significant difference when compared to the breast cancer patient and control group (p value < 0.05). Since the patients samples analysed during this study, also included 50 FFPE tissues, in addition to the genomic DNA extracted from blood, it was suspected that the genotypes for the studied SNPs may have altered in the tumor tissue as a result of the expansions in this tissue. Comparison of the genotypes for all four SNPs in the tumor tissue, with the genotype in the adjacent normal tissue from the same person, did not show any difference in all patients for three SNPs. Only one tissue of the 50 tissues samples (98%) investigated had a different genotype for one SNP in the tumor tissue compared to the normal tissue, and this may have occurred during the tumor genesis. It is expected that mutations during expansion in tumor tissue may alter genotype. However, in this study, we did not observe generation of a mutant of the four studied SNPs except one new variant for one SNP.

Interestingly, when comparing breast cancer tissues and blood from breast cancer, we found highly significant difference between the two groups for rs34404564 at genotypic levels, i.e., the heterozygosity AG and homozygosity GG frequency differences. The transition from A to G is protective against breast cancer development as shown when the patient’s results are compared with the controls. This result is interesting as it shows that even within the patients; the cancer tissue has less protective genotype and allele frequency possibly due to somatic mutation, compared to the germline mutation in the blood of the patients. Since this SNP is protective, somatic mutation is converting the mutated SNP (G) back to the wild-type (A), and hence a significantly lower frequency is noted in the cancer tissue, thus may predispose the tissue to malignant transformation. No significant differences in the rs78202224, rs35253356, and rs4977219 polymorphisms were observed between tissue patient and blood patient (results not shown).

Median age at diagnosis of breast cancer in Saudi Arabian patient is 48 years, while in the United States; it is substantially lower than 62 years [27]. There is no significant relationship between diagnosis at age less than 48 years and more than 48 years.

Of the four polymorphism investigated in breast cancer, rs78202224 showed that the G>T transversion increases the risk of breast cancer significantly in the total patient group. The risk remains to be significant in HER2-, ER- and PR- patients when compared with healthy control. The significance is lost in HER2+, ER+, and PR+ when compared with healthy control.

The minor allele of the SNPs rs35253356 and rs34404564 also showed a protective effect against the development of breast cancer, in the total patients, and the difference was statistically significant for the latter. The significant effect was seen in those >48 years, HER+, HER-, ER+ and PR+ patients, compared to the controls. The mechanism by which this protective effect occurs is unknown. The SNP rs4977219 showed no significant effect on breast cancer development in the total patients, or in the patients grouped according to age or hormonal status. The distribution of the genotypes in patients with high and intermediate severity, showed that there were some differences, where rs35253356 AG+GG were found in 66.7% of the patients with severe presentation, rs4977219 genotypes AC+CC were present in 83% of the patients with severe presentation and rs34404564 genotypes AA+AG in 66.7% of the patients with severe presentation. No significant differences were shown in genotypes of rs78202224. These preliminary results show that the genotypes of SNPs in HSF1 do influence disease presentation of breast cancer. Further detailed studies are required for further confirmation.

Heat shock factor 1 is regarded as an inactive monomer in the cytoplasm. Our results showed that HSF1 protein is expressed in the epithelial cells of the non-cancerous tissues, though at a low level, however, its expression is significantly increased in all breast cancer patients investigated in this study, as seen by IHC results. These results could not be confirmed by gene expression studies using qRT-PCR, since the tissues used were formalin fixed (FFPE) tissues. For the RNA extraction, tissue collected in RNA-later solution is necessary and was not available during the present study.

Positive staining for HSF1 was found in the nucleus and cytoplasm, albeit it was stronger in the nucleus than in the cytoplasm, a finding that confirms the main location of HSF1, which is in the nucleus [28]. Upon activation of HSF1, and homo trimerization step, it localizes to the nucleus and acts as a transactivator. Sentagata et al. [4] explained that nuclear HSF1 levels were increased in 80% of *in situ* and invasive breast carcinomas in over 1,800 women. HSF1 expression was linked to high histologic grade, larger tumor size, and nodal involvement at diagnosis in invasive carcinomas [4]. Overexpression of HSF1 has been reported in several cancer studies. Ishiwata et al. [29] reported that HSF1 protein expression was higher in primary oral squamous cell carcinoma compared to the normal oral tissue. Dudeja et al. [30] reported high HSF1 protein expression in human pancreatic cancer relative to the normal pancreatic tissues. Chen et al. [31] reported an increase level of HSF1 protein expression in human hepatocellular carcinomas. Hoang et al. [32] reported that in most prostate cancer human specimens, the level of HSF1 protein is up-regulated when the results were compared to normal prostate cancer tissue.

HSF1 is a transcriptional activation factor for HSP. Over-expression of HSF1 results in up-regulation of HSP especially HSP70, HSP90, and HSP27. HSP90 holds the main responsibility of enhancement of the spread of tumor via chaperoning the oncogenes that have mutated and over-expressed and leads to transformation and progression of tumors. HSP27 and HSP70 are involved in enhancing mammary tumorigenesis by inhibiting apoptosis and senescence.

Finally, it is suggested that the possible mechanism that causes increased susceptibility to cancer in presence of variant of rs78202224, may be due to its close vicinity to LZ, and may alter zipping ability of the LZ. On the other hand, rs34404564, which lies in the first intron of the *HSF1* gene, may be located in an area which influences splicing of the heterogenous RNA to form mRNA of HSF1 and has an influence on the expression of the *HSF1* gene. Further studies are warranted in order to clarify the underlying mechanism by which these two polymorphisms influence cancer development.

In conclusion, the present study shows that rs78202224 Pro-to-Thr (a missense mutation) is associated with increased breast cancer risk, breast cancer at TT+GT genotype and at T allele, it also show high risk in HER2 negative at TT+GG genotype and T allele, HER2 positive at TT+GG genotype, ER-negative at TT and TT+GG genotype and T allele, PR negative at TT+GG genotype and T allele at TT+GG genotype and T allele. rs34404564 polymorphism show protective effect in breast cancer patient at AG and GG+AG genotype, it also shows decreased risk in age > 48 at AG; GG; GG+AG genotype and G allele, HER2 negative at AG genotype, HER2 positive, ER positive and PR positive at AG and GG+AG genotype. Whereas no significant relationship was found between rs35253356 and rs4977219 polymorphism investigated in breast cancer cases compared to healthy individuals. On the other hand, the present study showed a high HSF1 protein expression in all invasive and *in situ* breast carcinoma while the expression was at a lower level in the normal tissue. Every histological grade examined also showed a high expression level. A stronger positive staining for HSF1 was found in the nucleus than in the cytoplasm.

## Supporting information

S1 File Age, hormonal status, tissue grade and genotyping results of the breast cancer patients (blood and tissue) and controls. (XLSX)
